# Additive insulinogenic action of Opuntia ficus-indica cladode and fruit skin extract and leucine after exercise in healthy males

**DOI:** 10.1186/1550-2783-10-45

**Published:** 2013-10-21

**Authors:** Louise Deldicque, Karen Van Proeyen, Monique Ramaekers, Ivo Pischel, Hartwig Sievers, Peter Hespel

**Affiliations:** 1 Exercise Physiology Research Group, Department of Kinesiology, KU Leuven, Leuven, Belgium; 2 PhytoLab GmbH & Co. KG, Vestenbergsgreuth, Germany

**Keywords:** Nutritional supplements, Post exercise recovery, Glucose clearance, Glycogen resynthesis, Endurance athletes, Amino acids

## Abstract

**Background:**

Oral intake of a specific extract of *Opuntia ficus-indica* cladode and fruit skin (OpunDia™) (OFI) has been shown to increase serum insulin concentration while reducing blood glucose level for a given amount of glucose ingestion after an endurance exercise bout in healthy young volunteers. However, it is unknown whether OFI-induced insulin stimulation after exercise is of the same magnitude than the stimulation by other insulinogenic agents like leucine as well as whether OFI can interact with those agents. Therefore, the aims of the present study were: 1) to compare the degree of insulin stimulation by OFI with the effect of leucine administration; 2) to determine whether OFI and leucine have an additive action on insulin stimulation post-exercise.

**Methods:**

Eleven subjects participated in a randomized double-blind cross-over study involving four experimental sessions. In each session the subjects successively underwent a 2-h oral glucose tolerance test (OGTT) after a 30-min cycling bout at ~70% VO_2_max. At t_0_ and t_60_ during the OGTT, subjects ingested 75 g glucose and capsules containing either 1) a placebo; 2) 1000 mg OFI; 3) 3 g leucine; 4) 1000 mg OFI + 3 g leucine. Blood samples were collected before and at 30-min intervals during the OGTT for determination of blood glucose and serum insulin.

**Results:**

Whereas no effect of leucine was measured, OFI reduced blood glucose at t_90_ by ~7% and the area under the glucose curve by ~15% and increased serum insulin concentration at t_90_ by ~35% compared to placebo (P<0.05). From t_60_ to the end of the OGTT, serum insulin concentration was higher in OFI+leucine than in placebo which resulted in a higher area under the insulin curve (+40%, P<0.05).

**Conclusion:**

Carbohydrate-induced insulin stimulation post-exercise can be further increased by the combination of OFI with leucine. OFI and leucine could be interesting ingredients to include together in recovery drinks to resynthesize muscle glycogen faster post-exercise. Still, it needs to be confirmed that such nutritional strategy effectively stimulates post-exercise muscle glycogen resynthesis.

## Background

High-intensity exercise typically leads to a depletion of body carbohydrate stores, primarily muscle glycogen [1]. Hence high-dose oral carbohydrate intake during recovery after exercise is pivotal to muscle glycogen resynthesis and thus repletion of carbohydrate stores [2]. Therefore, typical sports recovery drinks include a high carbohydrate dose (~50-100 grams), composed of rapidly absorbable sugars such as maltodextrin (a D-glucose polymer) and D-glucose (~dextrose). The increased plasma insulin level due to high-dose glucose ingestion is pivotal to stimulation of muscle glucose uptake and glycogen synthesis [3,4]. Insulin, which is secreted by the pancreatic β-cells upon elevated circulating glucose concentration, stimulates glucose import in muscle cells via the GLUT4 membrane protein. It also stimulates the incorporation of the glucose molecules into the glycogen molecule via activation of the glycogen synthase enzyme [5]. In this regard it is also important to note that muscular insulin sensitivity is markedly increased following muscle contractions [6]. Thus, any intervention that could elevate plasma insulin and/or further increase insulin sensitivity following exercise could facilitate repletion of muscle glycogen stores, and thus serve as a useful recovery agent.

In this respect, the addition of amino acids, and more particularly leucine, to a carbohydrate-rich drink is a frequent strategy used by athletes to increase insulin secretion and thereby enhance glycogen resynthesis. Leucine has a strong insulinotropic action which contributes to a faster glycogen resynthesis after exercise [7,8]. Based on recent reports [9,10], *Opuntia ficus-indica* intake could be another interesting nutritional strategy to stimulate insulin secretion and glycogen resynthesis after exercise. *Opuntia ficus-indica* is one of the approximately 200 species of the *Opuntia* genus, which belongs to the Cactaceae family [11]. *Opuntia ficus-indica* has been found to lower blood glucose and to increase basal plasma insulin levels in animals [9,12] as well as in humans [10,13,14]. This indicates a direct action on insulin secretion at the site of pancreatic β-cells rather than an indirect action via increased blood glucose levels.

Our group has recently shown that oral intake of a specific extract of *Opuntia ficus-indica* cladode and fruit skin (OFI) increases serum insulin concentration while reducing blood glucose level for a given amount of glucose ingestion after an endurance exercise bout in healthy young volunteers [10]. In a dose–response experiment we also found 1000 mg of OFI to cause a maximal increase of plasma insulin concentration. However, we did not evaluate the interaction of OFI with other insulinogenic agents like leucine. Moreover, commercial recovery drinks contain a maximal leucine dose of 3 g whereas only high doses (~7 g) have been shown to increase carbohydrate-induced insulin stimulation after exercise [7,8,15]. It is unknown whether lower doses of leucine increase carbohydrates-induced insulin stimulation as well. Against this background, the aims of the present study were: 1) to compare the degree of insulin stimulation by OFI with another prevailing strategy in sports nutrition to stimulate post exercise insulin release, i.e. 3 g oral leucine administration; 2) to determine whether OFI and leucine can have an additive action to elevate post exercise plasma insulin concentration.

## Methods

### Subjects

Eleven healthy, physically active males were included in the study (age: 21.1 ± 0.9 y; body weight: 74.5 ± 4.2 kg; VO_2_ max: 65 ± 4 ml·min^-1^·kg). After approval of the study protocol by the local Ethics Committee (KU Leuven), subjects were asked to give their written consent after they were informed of all experimental procedures and risks associated with the experiments. Furthermore, they were submitted to a medical screening before being enrolled in the study. Subjects who had any pathology or were taking any medication or nutritional supplements that were not compatible with the study protocol were excluded. All procedures were carried out in accordance with the Declaration of Helsinki (2000) of the World Medical Association.

### Preliminary testing

Two weeks before the start of the study, the subjects performed a maximal incremental exercise test (initial load 60 Watts (W) + 35 W per 3 min) on a bicycle ergometer (Avantronic Cyclus II, Leipzig, Germany) to determine the rate of maximal oxygen uptake (VO_2_max) and the corresponding workload. Heart rate (Polar, Kempele, Finland), VO_2_ and VCO_2_ (Cortex Metalyzer II, Leipzig, Germany) were continuously measured during the test.

### Study protocol

A double–blind randomized cross-over study was performed. Subjects participated in four experimental sessions with a 1-week interval in between. Subjects abstained from any high intensity exercise for 48 hours prior to the experiments. In the evening before the experimental sessions, subjects received a standardized carbohydrate-rich dinner (860 kcal, 73% carbohydrates, 14% fat, 13% protein), after which they remained fasted. On the morning of the experiments they reported to the laboratory in the fasted state between 8:00 and 9:00a.m. to perform a 30-min endurance exercise bout at 70% of their previously determined VO_2_max at a cadence fixed at 90-100 rpm. At the end of the exercise the subjects got seated in a comfortable armchair and an intravenous catheter was inserted into an arm vein for repeated blood sampling during the experiment after which a baseline blood sample was collected. The subjects received capsules containing either: 1) LUVOS Heilerde serving as placebo (PL); 2) 1000 mg *Opuntia ficus-indica* cladode and fruit skin extract (OFI) (OpunDia™, Finzelberg, Germany); 3) 3 g leucine (LEU) (Ajinomoto, Japan); 4) 1000mg *Opuntia ficus-indica* extract + 3 g leucine (OFI+LEU). All capsules had identical appearance and the number of capsules ingested was the same for each condition. OpunDia™ is a preferred blend of *Opuntia ficus-indica* cladode and fruit skin extract containing 75% cladode extract and 25% fruit skin extract (for both extraction solvent: water; DER (drug-to-extract ratio) 2–4:1; 50% native extract, 50% collagen hydrolysate as excipient). All plant materials of *Opuntia ficus-indica* were obtained from a US American-based cultivation having breeding experience since 1923 [16]. Voucher specimens of the drug material are deposited at PhytoLab, Vestenbergsgreuth, Germany. The dose of 1000 mg OFI was selected based on preliminary dose–response data showing 1000 mg to be the lowest dose needed to maximally increase plasma insulin concentration [10]. After ingestion of the supplement together with a 75 g glucose bolus in 300 ml water, a 2-hr oral glucose tolerance test (OGTT) was started at time 0 (t_0_). Thereafter, a blood sample (5 ml) was collected from the arm vein catheter into vacuum tubes containing Silica clot activator (BD Vacutainer, NJ, USA), at 30, 60, 90, and 120 min. During the OGTT, an additional dose of OFI (1000 mg) and/or LEU (3 g), together with glucose (75 g), was given at t_60_ to maintain blood glucose concentration high. Blood samples were centrifuged (1500 rpm for 15 min at 4°C) to spin down the serum which was stored at −80°C until analyzed at a later date for insulin.

### Blood samples

Serum insulin was assayed by chemiluminescence using the Siemens DPC kit and according to the instructions by the manufacturer. Blood glucose concentration was determined on 10 μl blood coming from the earlobe using an automated micro-analyzer (Arkray Inc., Kyoto, Japan).

### Data calculations and statistical analyses

The positive incremental area under the glucose curve and the insulin curve were calculated as previously described [17,18]. The differences between the conditions (PL, OFI, LEU and OFI+LEU) were analyzed by Student’s paired T-tests using the SigmaPlot® statistical software package. A probability level of P≤0.05 was considered statistically significant. All data are expressed as means ± SE.

## Results

### OFI and leucine have an additive insulinogenic effect

**Figure 1:**
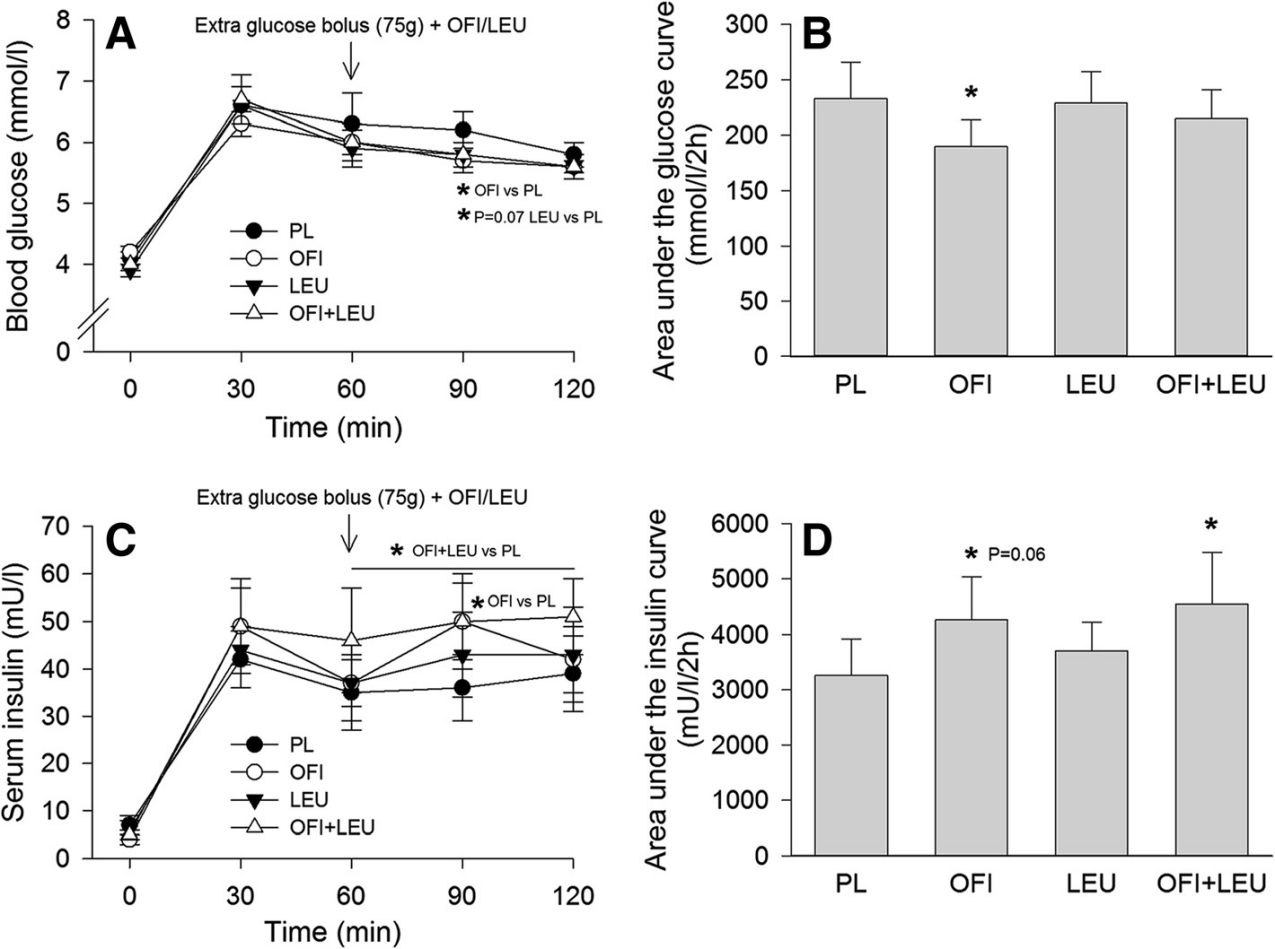
**Effect of *Opuntia ficus-indica *cladode and fruit skin extract and/or leucine on blood glucose and serum insulin during a post exercise OGTT.** Concentrations of blood glucose **(A)** and serum insulin **(C)**, as well as the calculated area under the curve for blood glucose **(B)** and serum insulin **(D)** during a 120-min OGTT after exercise and after having ingested a placebo (PL), *Opuntia ficus-indica* cladode and fruit skin extract (OFI), leucine (LEU) or *Opuntia ficus-indica* cladode and fruit skin extract + leucine (OFI+LEU). Data are means ± SE (n=11). *P<0.05 vs PL.

All subjects tolerated the supplements well and none exhibited symptoms of gastrointestinal distress. Post exercise blood glucose concentration was 4.0 ± 0.1 mmol/l in all experimental conditions (Figure 1A). Thirty minutes following the initial 75 g glucose bolus together with the supplement(s), blood glucose peaked at 6.6 ± 0.1 mmol/l, to gradually decrease thereafter. Compared with PL, OFI reduced blood glucose at t_90_ by 7% (5.7 ± 0.2 in OFI vs 6.2 ± 0.3 mmol/l in PL, P<0.05, Figure 1A) and the area under the 2-h glucose curve by about 15% (190 ± 24 in OFI vs 233 ± 33mmol/l/2h in PL, P<0.05, Figure 1B). Leucine tended to decrease blood glucose concentration at t_90_ (P=0.070, Figure 1A). Post exercise serum insulin concentration was 5.7 ± 0.6 mU/l and reached 35-50 mU/l during the OGTT depending on the treatment. From t_60_ to the end of the OGTT, serum insulin concentration was higher in OFI+LEU than in PL (P<0.05, Figure 1C). OFI alone increased insulin concentration only at t_90_ (50 ± 10 in OFI vs 36 ± 7 mU/l in PL, P<0.05). Accordingly, OFI+LEU increased by about 40% (4555 ± 923 in OFI+LEU vs 3259 ± 663 mU/l/2h in PL, P<0.05) and OFI alone tended to increase (4272 ± 761 in OFI vs 3259 ± 663 mU/l/2h in PL, P=0.059) the area under the insulin curve (Figure 1D). Leucine had no effect on insulin concentration.

## Discussion

In a recent study, we showed for the first time that OFI can elevate circulating plasma insulin concentration during high rate carbohydrate ingestion in humans at rest and after exercise [10]. This finding is particularly relevant to endurance athletes seeking to restore high muscle glycogen concentration between training sessions so as to maintain training quality [19]. As muscle glycogen repletion is sensitive to insulin [3], most prominently during the initial hours following an exercise bout [20,21], it is important for athletes to establish high circulating plasma insulin concentrations during early recovery following a strenuous training. It is of note that muscle insulin sensitivity is enhanced after exercise, which facilitates glycogen resynthesis compared with rest [6]. High rate carbohydrate ingestion, up to 1.0-1.2 g/kg/h for a few hours, is the prevailing nutritional strategy to increase glucose delivery to muscles together with elevated plasma insulin concentration and thereby stimulate glycogen resynthesis [7,22]. Adding proteins to a carbohydrate load will even speed up glycogen repletion due to the insulinogenic action of proteins and more particularly due to the branched-chain amino acid leucine [7,8,15]. Adding 0.4 g casein hydrolysate/kg/h to a drink containing 0.8 g carbohydrates/kg/h more than doubled plasma insulin response compared with only the carbohydrates. Insulin response was even tripled when 0.1 g leucine/kg/h was added to the carbohydrates/casein hydrolysate drink [15]. Similar results were obtained previously, but in those earlier studies both leucine and phenylalanine were added to the supplements, which makes it impossible to isolate the actions of the two amino acids [7,8]. In the study by Kaastra [15], drinks were not isoenergetic, which may account for the difference in plasma insulin concentration. However, when drinks were prepared to be isocaloric, carbohydrates combined with proteins still induced a higher insulin response than carbohydrates alone [7]. Contrary to those previous studies, our results do not show a clear additional insulinogenic effect of leucine when co-ingested with a high amount of carbohydrates. We deliberately chose a dose of 3 g of leucine instead of ~ 7 g (0.1 g/kg) by others [7,8,15] because leucine has a very low palatability, and therefore most commercial sports supplements contain a maximum of 3 g leucine. We also based our decision on a recent report showing that 3.42 g leucine alone, in the absence of carbohydrate intake and at rest, increased plasma insulin concentration by 50% within 30 min before returning to basal levels [23]. It is thus possible that the smaller amount of leucine, compared to previous studies, added to the high amount of glucose (~1 g/kg/h) was not large enough to further enhance plasma insulin concentration in the present study.

Based on our data, 1000 mg OFI had a slightly higher insulinogenic action than 3 g leucine, certainly 30 min after ingestion of the glucose + OFI beverage. OFI seems to stimulate insulin production acutely and rapidly as serum insulin concentrations during the OGTT each time were increased 30 min after OFI ingestion but no more 60 min after OFI intake. The insulinogenic action of OFI thus clearly is short-lived. The largest effect on plasma insulin concentration was obtained by the combined ingestion of OFI plus leucine. Indeed, insulin concentration was persistently elevated during the second hour of the OGTT when OFI and leucine were administered together. In addition, a trend (P=0.09) to increase in insulin concentration was observed in OFI + LEU compared with OFI alone at 60 and 120 min. As blood glucose concentrations were not modified by OFI plus leucine, the increase in insulin did not result from higher blood glucose levels. Our results rather indicate that OFI and leucine directly stimulate pancreatic insulin release, and that the effects of both agents are additive. Whereas the physiological mechanism by which OFI facilitates glucose-induced pancreatic insulin release remains to be elucidated, it is known that leucine increases pancreatic β-cell insulin secretion through: 1) its oxidative decarboxylation; 2) its ability to allosterically activate glutamate dehydrogenase, and 3) its transamination to α-ketoisocaproate [24]. Those events will subsequently lead to an increased tricarboxylic acid-cycle flux, an increased ATP/ADP ratio, the closure of the ATP-sensitive potassium channels, a depolarization of the plasma membrane and the opening of the calcium sensitive channels which will finally cause the secretion of insulin [25-27]. Whether OFI increases the tricarboxylic acid-cycle flux in beta-cells as well, or whether it depolarizes the membrane via a different mechanism than leucine remains to be investigated.

The combination of OFI with leucine seems the best option to increase plasma insulin concentrations after exercise and thereby to potentially accelerate glycogen resynthesis. Nevertheless we did not measure any difference in the area under the glucose curve when both treatments were given together compared to placebo, which could indicate that muscle glucose uptake probably is not substantially modified by combined OFI plus leucine administration. Blood glucose concentration during an OGTT reflects the equilibrium between the rate of glucose appearance from the gut and liver, and the rate of disappearance through peripheral glucose uptake. It can be hypothesized that OFI combined with leucine actually increased both processes that resulted in unchanged blood glucose concentrations. However, this is not likely to be the case as the addition of amino acids to a carbohydrate-rich drink was previously shown to decrease the rates of appearance and disappearance of blood glucose instead [15]. As the decreases were equal in amplitude, it was suggested that amino acids-induced insulin stimulation accelerates glycogen resynthesis after exercise by increasing glycogen synthase activity rather than by increasing muscle glucose uptake [15]. Further studies should try to determine whether the higher circulating insulin levels established by combined OFI plus leucine administration together with high rate glucose uptake post exercise, effectively translate into higher glycogen synthase activity and glycogen resynthesis rate following exercise.

## Conclusion

Carbohydrate-induced insulin stimulation after exercise can be further increased by the combination of *Opuntia ficus-indica* cladode and fruit skin extract with leucine. In the perspective of developing optimal nutritional strategies to recover muscle glycogen faster after high-intensity endurance exercise, OFI and leucine could be interesting ingredients to include together in recovery drinks. Still, it needs to be confirmed that such nutritional strategy effectively stimulates post exercise muscle glycogen resynthesis.

## Abbreviations

GLUT4: Glucose transporter 4; LEU: Leucine; OFI: *Opuntia ficus-indica* cladode and fruit skin extract; OGTT: Oral glucose tolerance test; VO2max: Maximal oxygen uptake.

## Competing interests

Ivo Pischel and Hartwig Sievers are employees of PhytoLab GmbH & Co. KG, Germany and were involved in the study design, but not in any data generation or processing. OpunDia™ is applied for patents by Finzelberg GmbH & Co. KG, Germany, e. g. US 2010323045 (A1) - Extract Formulation of Opuntia ficus Indica (Priorities: US20080741562 20081106; EP20070120081 20071106; US20070002058P 20071106; WO2008EP65048 20081106).

## Authors’ contributions

PH, IP and HS were responsible for the concept of this project and for the study design. KVP, and MR were responsible for the acquisition and the analysis of the data. PH, KVP and LD were responsible for the interpretation of the data. PH and LD wrote the first version of the manuscript which was edited by the other authors. The final version was approved by all authors.
